# The Modulatory Role of CYP3A4 in Dictamnine-Induced Hepatotoxicity

**DOI:** 10.3389/fphar.2018.01033

**Published:** 2018-09-19

**Authors:** Zhuo-Qing Li, Li-Long Jiang, Dong-Sheng Zhao, Jing Zhou, Ling-Li Wang, Zi-Tian Wu, Xian Zheng, Zi-Qi Shi, Ping Li, Hui-Jun Li

**Affiliations:** ^1^State Key Laboratory of Natural Medicines, China Pharmaceutical University, Nanjing, China; ^2^Affiliated Hospital of Integrated Traditional Chinese and Western Medicine, Nanjing University of Chinese Medicine, Nanjing, China; ^3^Jiangsu Province Academy of Traditional Chinese Medicine, Nanjing, China

**Keywords:** Dictamni Cortex, dictamnine, liver injury, CYP3A4, induction/inhibition effect

## Abstract

Dictamni Cortex (DC) has been reported to be associated with acute hepatitis in clinic and may lead to a selective sub-chronic hepatotoxicity in rats. Nevertheless, the potent toxic ingredient and the underlying mechanism remain unknown. Dictamnine (DTN), the main alkaloid from DC, possesses a furan ring which was suspected of being responsible for hepatotoxicity via metabolic activation primarily by CYP3A4. Herein, the present study aimed to evaluate the role of CYP3A4 in DTN-induced liver injury. The *in vitro* results showed that the EC_50_ values in primary human hepatocytes (PHH), L02, HepG2 and NIH3T3 cells were correlated with the CYP3A4 expression levels in corresponding cells. Furthermore, the toxicity was increased in CYP3A4-induced PHH by rifampicin, and CYP3A4 over-expressed (OE) HepG2 and L02 cells. Contrarily, the cytotoxicity was decreased in CYP3A4-inhibited PHH and CYP3A4 OE HepG2 and L02 cells inhibited by ketoconazole (KTZ). In addition, the hepatotoxicity of DTN in enzyme induction/inhibition mice was further investigated in the aspects of biochemistry, histopathology, and pharmacokinetics. Administration of DTN in combination with KTZ resulted in attenuated liver injury, including lower alanine transaminase and aspartate transaminase activities and greater AUC and *C*_max_ of serum DTN, whereas, pretreatment with dexamethasone aggravated the injury. Collectively, our findings illustrated that DTN-induced hepatotoxicity correlated well with the expression of CYP3A4, namely inhibition of CYP3A4 alleviated the toxicity both *in vitro* and *in vivo*, and induction aggravated the toxicity effects.

## Introduction

There are increasing applications of Chinese herbal medicines (CHMs) for the prevention and treatment of various illnesses, and in parallel, the increased use has been accompanied by an emerging concern regarding the safety of CHMs (Zeng and Jiang, 2010; Willamson et al., 2013; Wang et al., 2015). Cases of severe adverse effects caused by CHMs, particularly hepatotoxicity, have been frequently reported over the past few decades (Teschke et al., 2014; Chan et al., 2015; Coghlan et al., 2015). Several CHMs have been identified as a major cause of drug-induced liver injury, namely herb-induced liver injury (HILI), for example, Polygoni Multiflori Radix (He-Shou-Wu in Chinese) was pointed out to use with caution for the severe hepatotoxicity (Lin et al., 2015; Li H. et al., 2016). Undoubtedly, it is important to pay attention to the health risk of CHMs.

**FIGURE 1:**
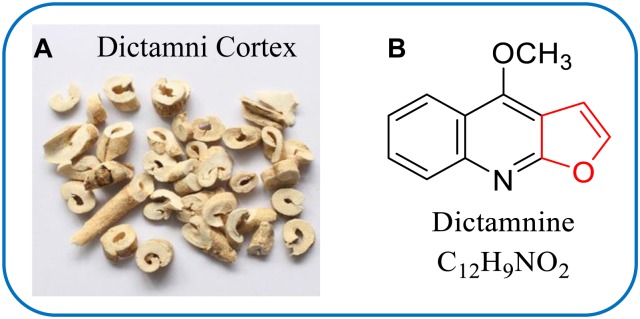
Dictamni Cortex **(A)** and chemical structure of dictamnin (DTN) **(B)**.

Dictamni Cortex (DC, Bai-Xian-Pi in Chinese) (**Figure 1A**) is the root bark of *Dictamnus dasycarpus* Turcz. (Family Rutaceae), which is extensively spread throughout China and has been used as a CHM for the treatment of rheumatism, jaundice, skin diseases and chronic hepatitis with a long history (Du et al., 2005; Lv et al., 2015; Chang et al., 2016). However, the safety of DC has been questioned as exposure to DC might be associated with the risk of hepatitis in clinic (Blackwell, 1996; Efferth et al., 2017). A systematic review in Korea reported that 37.1% of identified HILI cases were attributed to DC (Lee et al., 2015). Additionally, an investigation in mice and rats showed that oral administration of DC aqueous extract led to sub-chronic toxicity with alterations of hematological and liver function parameters (Wang et al., 2014). Although chemical studies had been extensively conducted and a large number of compounds, including quinolone alkaloids (Du et al., 2005; Sun et al., 2013), furoquinoline alkaloids (Alkhmedzhanova et al., 1978; Lv et al., 2015) and limonoides (Sun et al., 2015), were isolated in DC, the hepatotoxicity-related causal ingredients remain unidentified so far.

It is widely accepted that cytochrome P450 (CYPs)-mediated metabolic activation is responsible for the hepatotoxicity of some natural products (Njuguna et al., 2012; Ruan et al., 2014; Lin et al., 2016) via the formation of reactive electrophilic intermediates that react with nucleophilic sites of macromolecules, such as protein (Xia et al., 2016), glutathione (Jiang L.L. et al., 2017), and nucleic acid (Yu et al., 2014). Among those potential toxic natural products, furan-containing compounds are representatives that are prone to be bioactivated into an epoxide or a *cis*-enedione intermediate, which could bind with cellular proteins and thereby trigger toxicity (Peterson, 2013). In this aspect, a striking example was disobulbin B, a furanoid diterpenoid lactone isolated from *Dioscorea bulbifera* L., which was proven to be hepatotoxic through metabolic activation of the furan moiety in the presence of CYP 3A (Li W. et al., 2016). Consequently, the furan ring moiety is considered to be an alert structure for medicinal chemists and risk assessors (Kalgutkar et al., 2005).

Given that dictamnine (DTN, **Figure 1B**), a major furoquinoline alkaloid component in DC, possesses a furan ring in its molecular structure, we hypothesized that DTN might be potentially responsible for DC-induced hepatotoxicity. Actually, Guo et al. (2013) found that DTN damaged the mitochondrial membrane and exerted cytotoxicity. More importantly, it was confirmed that DTN was predominantly metabolized into an epoxide intermediate (2,3-epoxide) *in vitro*, which was primarily in the participation of CYP3A4 (Wang et al., 2016). Furthermore, the epoxide intermediate could subsequently conjugated with *N*-acetylcysteine to form conjugate with CYP3A4 was primarily responsible for the metabolic activation process (Feng et al., 2016). Thus, evidences have shown the link between the metabolic activation of DTN and CYP3A4, namely CYP3A4 was the predominant enzyme involved in the metabolism of DTN. However, the correlation between CYP3A4-mediated metabolism and DTN-induced hepatotoxicity was not clear.

**FIGURE 2:**
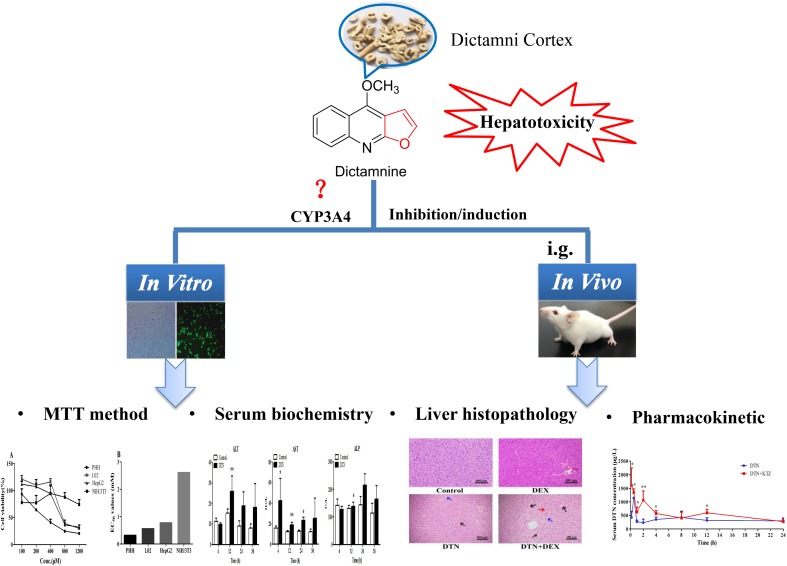
Scheme for the study. ^∗^*p* < 0.05, ^∗∗^*p* < 0.01 vs. control group.

Therefore, in an attempt to define the correlation, we evaluated the toxicological and histopathological responses with CYP3A4 inducer and inhibitor. The correlation was unveiled by comparison studies at three levels: (1) cytotoxicity of DTN in different CYP3A4 expression cells, including primary human hepatocytes (PHH) with CYP3A4 inducer or inhibitor, NIH3T3 cells, wild type (WT) HepG2 and L02 cells, and stable CYP3A4 over-expressed (OE) HepG2 and L02 cells with CYP3A4 inducer or inhibitor; (2) biochemical and histopathological differences in DTN-induced hepatotoxicity with or without CYP3A4 induction/inhibition in mice; (3) differences of pharmacokinetic parameters between CYP3A4 inhibition groups and non-pretreatment groups in mice. Based on the obtained results of this study, we confirmed the vital modulatory role of CYP3A4 in DTN-associated hepatotoxicity. **Figure 2** displayed the overall research strategy for this study.

## Materials and Methods

### Reagents and Materials

Dictamnine and γ-fagarine (>98% purity) were purchased from Chengdu Must Biotechnology Co., Ltd. (Chengdu, China). Dexamethasone (DEX), rifampicin (RIF), ketoconazole (KTZ), diclofenac sodium (DCF), corn oil, CMC-Na and 3-(4,5-dimethyl-2-thiazolyl)-2,5-diphenyl-2-H-tetrazolium bromide (MTT) were products of Aladdin Chemical Co., Ltd. (Shanghai, China). A neutral red kit was procured from Beyotime Biotechnology Co., Ltd. (Shanghai, China). Gibco^®^ high glucose Dulbecco’s modified Eagle’s medium (DMEM) and lipo2000 were obtained from Thermo Fisher Scientific Technology Co., Ltd. (Shanghai, China). Medium for PHH was purchased from Zhong Qiao Xin Zhou Biotechnology Co., Ltd. (Shanghai, China). A PCR kit was purchased from Vazyme Biotech Co. Ltd. (Nanjing, China). All other chemical reagents were from Merck Life Science Co., Ltd. (Shanghai, China).

### Animals and Treatment

Male adult ICR mice (20 ± 2 g) were supplied by the Model Animal Research Center of Nanjing University (Nanjing, China). All experiments and animal care were conducted in accordance with the Provision and General Recommendation of Chinese Experimental Animals Administration Legislation and were approved by the Science and Technology Department of Jiangsu Province [license number: SYXK (SU) 2016-0011]. The mice were housed in a controlled temperature and humidity of 22 ± 2°C and 55 ± 5%, respectively, and maintained on a 12 h light/dark cycle with free access to food and water. Mice were fastened for 12 h and free to water before treatment.

Mice were randomly assigned into two groups for different treatment. The protocol was conducted as previously described with minor modification (Iida et al., 2015; Li W. et al., 2016). The mice in group A (*n* = 4) were pretreated with DEX (70 mg/kg, dissolved in corn oil) or vehicle by intraperitoneal (*i.p.*) injection for 3 consecutive days, and 1 h after the last injection, a single dose of DTN (640 mg/kg, suspended in 0.5% CMC-Na) or vehicle was orally administrated to the mice. Mice in group B (*n* = 4) were treated with DTN (320 mg/kg) for 4 consecutive days, and 1.5 h before the last administration of DTN (640 mg/kg) on the 5th day, mice were treated with KTZ (75 mg/kg, dissolved in corn oil) or vehicle by *i.p.* injection. Blood and liver tissues were collected in 24 h following treatment. All the livers were quickly removed and the left lateral liver lobes were used for histopathological examinations.

### Cell Culture

Cryo-preserved PHH were supplied by the Zhong Qiao Xin Zhou Biotechnology Co., Ltd. (Shanghai, China; Lot No. 24595), and cultured in the supporting complete medium. HepG2, L02, and NIH3T3 cells were preserved in the State Key Laboratory of Natural Medicines (Nanjing, China). HepG2 and L02 cells, including CYP3A4 OE cells, were maintained in DMEM medium containing 10% fetal bovine serum, 100 units/mL penicillin and 100 μg/mL streptomycin, while NIH3T3 cells were cultured in DMEM with 10% calf serum instead of fetal bovine serum. Prior to any treatment, cells were trypsinized and plated in a decent destiny with the corresponding media at 37°C in an incubator with a humidified atmosphere of 5% CO_2_/95% air.

### Construction of Stable CYP3A4 OE HepG2 and L02 Cell Lines

The 1512-bp fragment containing the human CYP3A4 coding sequence plasmid (pCMV3-CYP3A4-GFPspark) was obtained from Sino Biological Inc. (Beijing, China). Cells were seeded into 100-mm culture dishes at a density of 1 × 10^6^ cells, and 24 h later cells were transfected with the above plasmid (1000 ng of DNA per dish) and lipo2000 according to the manufacturer’s instruction. The successful transfected cells expressed green fluorescent protein (GFP) and showed green fluorescence under fluorescence microscope (Nikon Corp., Tokyo, Japan). 24 h later, the medium was changed with hygromycin B (50 mg/mL) every 3rd day to a final concentration of 400 and 500 μg/mL for HepG2 and L02, respectively. Cells were allowed to grow in the selection medium for 4 weeks, at which time individual clonal expansions (clones) were visible. Each clone was transferred and tested for enzymatic activity by a quantitative polymerase chain reaction (Q-PCR), western blot analysis and determining hydroxylation of testosterone. DCF, a known hepatotoxic drug through metabolic activation by CYP3A4 (Shen et al., 1999; Boerma et al., 2012) was chosen as the positive control to verify the effect of CYP3A4 in the WT and OE cells.

### Characterization of CYP3A4 Enzyme Activity in HepG2 and L02 Cells

The experiment was conducted referring to Jiang J.Z. et al. (2017) with modification. Briefly, the CYP3A4 over-expressed (OE) and WT HepG2 and L02 cells were seeded into a six-well plate with a density of 8 × 10^4^ to 1 × 10^5^ cells/per well. Cells were incubated with the substrate testosterone (30 μM), which was from Shanghai Yuanye Biotechnology Co., Ltd. (Shanghai, China). The reaction was terminated by 2.4 mL of ice-cold acetonitrile containing an internal standard (9-aminoacridine, IS1) after 48 h exposure. The supernatant was collected, centrifuged at 12,000 × *g* for 10 min. 3.5 mL of supernatant was collected and dried (GeneVac EZ-2, England). The residue was reconstituted with 10% methanol (100 μL), centrifuged at 12,000 × *g* for 10 min. The supernatant was collected and 2 μL was to be analyzed with method A by liquid chromatography-tandem mass spectrometry (LC-MS/MS).

### Cytotoxicity Evaluation of PHH, HepG2, L02, and NIH3T3 Cells

The experiment in PHH was divided into two groups: cells were seeded at respective cell densities of 5 × 10^3^ and 2 × 10^3^, and then incubated for 24 h. The first group was incubated with KTZ (10 μM) for 2 h before DTN exposure (Kittler et al., 2014), and another group was pre-incubated with RIF (20 μM) for 72 h (Zahno et al., 2011). After that, cells were treated with gradient concentrations of DTN, DCF, and DMSO (solvent control) for 24 h. All of the compounds were dissolved in DMSO with the final concentration of DMSO was less than 0.2% in the incubation system. The MTT assay was employed and the optical density (OD) value was measured at 490 nm with a microplate reader (BioTek Instruments, Winooski, VT, United States).

The neutral red assay was performed to assess cell viability of NIH3T3 cells. Briefly, cells were seeded into a 96-well plate at a density of 8 × 10^3^ cells per well, incubated for 12 h, and then exposed to DTN and DMSO. After incubation, the neutral red reagent was added following the manufacturer’s instruction. The OD value was detected at 570 nm. The WT and CYP3A4 OE HepG2 and L02 were treated in the same manner as NIH3T3 cells, and cell viability was measured by MTT assay. Cell viability was calculated with the following equation (Eq. 1). OD value is presented as the mean ± SD from three independent replicate experiments.

$$\text{Cell viability }(\%)=\frac{\text{ODdrug-ODsolvent control}}{\text{ODblank control-ODsolvent control}}\times 100\%$$

### Q-PCR of WT, CYP3A4 OE HepG2 and L02 Cells, and Mice Liver Tissues

HepG2 and L02 cells (5 × 10^4^) were seeded and cultured in six-well plates. The success of transduction was assessed by fluorescence microscope. Thereafter, mRNA expressions of human CYP3A4 in the CYP3A4 OE HepG2 and L02 cells were determined by Q-PCR. Total RNA was extracted with TRIzol reagent (Vazyme Biotech Co., Ltd., Nanjing, China). The RNA quality was assessed with a Thermo Nanodrop Analyzer and only RNAs with OD_260_/OD_280_ ranging from 1.8 to 2.0 were used for further experiments. The primers (**Table 1**) were from Genscript Biotech Co., Ltd. (Nanjing, China), and reserve transcription was performed with a kit (Vazyme Biotech Co., Ltd., Nanjing, China) according to the manufacture’s instruction. The reverse reactions with a system containing 1000 ng of RNA were performed at 25°C for 10 min, 50°C for 30 min and 85°C for 5 min. PCR was performed on a Light Cycle 96 Real-time PCR system (Roche, Basel, Switzerland). In addition, 20 mg of each mouse liver sample was weighed, and homogenized with 1 mL TRIzol at 60 Hz for 30 s in 4°C. The supernatant was separated to extract the RNA. Other procedures were the same as described except that mouse primers were used (**Supplementary Table S1**). Human and mouse glyceraldehyde-3-phosphate dehydrogenase (GAPDH) were used as control.

**Table 1:** Sequences of primer pair used for PCR amplification.

Target genes	5′ → 3′ primer sequence (Forward)	5′ → 3′ primer sequence (Reverse)
hGAPDH	CCATGTTCGTCA TGGGTGTGAAC	GCCAGTAGAGGC AGGGATGATGTTC
hCYP3A4	CCTCCCTGAAAG ATTCAGCA	ATGAGAGCAAAC CTCATGCC


### Western Blot Analysis

Cells were lysed with lysis buffer, and then heated at 99°C in a metal bath for 30 min to denature proteins. After separation of protein (equal loading for each sample) by 10% sodium dodecyl sulfate-polyacrylamide gel electrophoresis (SDS-PAGE), 100 μg of protein samples were electrophoretically transferred onto a polyvinylidene difluoride (PVDF) membrane at 250 mA for 60 min. After blocking with phosphate buffered saline containing 0.1% Tween–20 (PBST) and 5% fat-free milk for 1 h, PVDF membranes were immunoblotted overnight at 4°C with rabbit anti-GFP tag (1:1000 dilution) (ABclonal Biotech Co., Ltd., Boston, MA, United States) and anti-actin (1:1000 dilution), followed by incubation with rabbit secondary horseradish peroxidase conjugated antibody (1: 2000 dilution) for 1 h at room temperature. After washing the membranes three times with PBST, immunoreactive bands were visualized via a Tanon Western blot detection system (Tanon Science and Technology Co., Ltd., Shanghai, China). Human β-actin was used as a loading control.

### Biochemical Assay and Histopathological Examinations

The collected blood samples were allowed to clot at room temperature, followed by centrifugation at 4°C, 1,000 × *g* for 10 min. The resulting sera were immediately sent for alanine transaminase (ALT) and aspartate aminotransferase (AST) assays to the laboratory in Jiangsu Province Hospital of Traditional Chinese and Western Medicine (Nanjing, China) on ice. The testing laboratory was authenticated with ISO15189 and the assay was performed in a Roche Cobas 8000 (ISE/701/701/502) automatic biochemical analyzer. Liver tissues were immediately fixed in 4% paraformaldehyde for at least 24 h, then paraffin-processed, and sectioned into 3-μm thick slices. The sections were stained with hematoxylin and eosin (H&E), and finally evaluated by a pathologist using an optical microscope.

### Pharmacokinetic Study

Mice (*n* = 4 for each time point) were orally treated with DTN (320 mg/kg, suspended in 0.5% CMC-Na) for 4 consecutive days, and 1.5 h before the last administration of DTN (640 mg/kg) on the 5th day, mice were treated with KTZ (75 mg/kg, dissolved in corn oil) or vehicle by *i.p.* injection. Blood samples were collected by cardiac puncture at time intervals of 0.16, 0.5, 1, 2, 4, 6, 8, 12, and 24 h after DTN administration. Aliquots (50 μL) of sera were mixed with 400 μL methanol containing γ-fagarine (final concentration was 20 ng/mL) as the IS2. The resulting mixture was vortex-mixed and centrifuged at 16,000 × *g* for 10 min to remove precipitated protein. The resulting supernatants were analyzed in method B by LC-MS/MS. A non-compartmental model was utilized to describe the serum concentration-time profiles of DTN. Pharmacokinetic parameters, including maximum serum concentration (*C*_max_), time to reach maximum serum concentration (*T*_max_), area under the serum concentration-time curve (AUC) and *t*_1/2_ were determined with DAS software (Version 2.0, Mathematical Pharmacology Professional Committee of China, Shanghai, China).

### LC-MS/MS Analysis

Analyses were conducted on an Agilent series 1260 LC system and an Agilent 6420 QqQ mass spectrometer (Agilent Technologies, Santa Clara, CA, United States). Method A was as follows: The separation was carried out on a Waters Cortecs column (2.1 mm × 150 mm, 1.6 μm) at 35°C. Acetonitrile and water containing 0.1% (*v/v*) formic acid was adopted as the mobile phase, the flow rate was 0.3 mL/mi. The gradient was as follows: 0–3 min, 8–25% B; 3–5 min, 25–70% B; 5–7 min, 70–8% B. Method B was as follows: The separation was carried out on an Agilent Poroshell 120 SB-C_18_ column (4.6 mm × 75 mm, 2.7 μm) at 30°C. Methanol/water (75/25, *v/v*) containing 0.1% (*v/v*) formic acid was adopted as the mobile phase, the flow rate was 0.3 mL/min, and the injection volume was set at 2 μL.

The mass spectrometer was operated in positive ion mode with electrospray ionization source. Quantitation was performed by multiple reaction monitoring mode. The characteristics of ion pairs, corresponding fragmentor and collision energy in method A for 6β-hydroxyl testosterone (6β-OHTST) were *m/z* 305 → 269 (130 V, 12 V), and those for corresponding IS1 were *m/z* 195 → 151 (120 V, 50 V); while the parameters in method B for DTN were *m/z* 200 → 185 (135 V, 35 V), and those for corresponding IS2 were *m/z* 230 → 200 (135 V, 30 V). Data collection and processing were conducted with MassHunter Workstation 07.00 (Agilent Technologies, Santa Clara, CA, United States).

### Statistical Analysis

All data are expressed as the mean ± SD. Figures were obtained by the GraphPad Prism 5 (GraphPad Software, Inc., San Diego, CA, United States). Statistical analyses were performed using SPSS 19 (IBM Corp., Albany, NY, United States). Differences between many groups at one level were tested by one-way ANOVA with Bonferroni’s *post hoc* test if the data were normally distributed. In the case of non-normally distributed data, Tamhane’s T2 statistics was performed. Differences between two groups were carried out using a two-tailed Student’s *t*-test. In all cases, a *p*-value less than 0.05 was considered to be significant.

## Results

### Cytotoxicity of DTN in PHH, HepG2, L02, and NIH3T3 Cells

**FIGURE 3:**
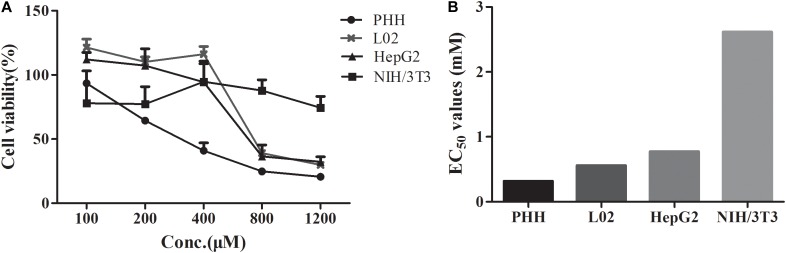
Cytotoxicity of DTN in primary human hepatocytes (PHH), L02, HepG2 for 24 h and NIH3T3 cells for 72 h **(A)**. EC_50_ values in different cells **(B)**. Data are represented as mean ± SD (*n* = 3).

The EC_50_ values of DTN between hepatocytes and non-hepatocytes NIH3T3 cells were compared. As shown in **Figures 3A,B**, the EC_50_ value in PHH was 0.32 mM, which was minimal, and the values in L02 cells (0.56 mM) and HepG2 cells (0.77 mM) were approximative. Nevertheless, the EC_50_ value in NIH3T3 cells (incubation for up to 72 h) was 2.6 mM, which was 8.3, 3.4, and 4.7 times higher than that in PHH, HepG2, and L02 cells, respectively. Meanwhile, the cell inhibition ratio was marginal in NIH3T3 cells after DTN treatment (100–1200 μM) with cell viabilities ranging from 77.9 to 94.7%. Hence, DTN was unable to induce cytotoxicity in NIH3T3 cells with free CYP3A4 expression. The above results indicated that DTN exhibited varied cytotoxicity in diverse cells with different CYP3A4 expression levels.

### The Effect of Inhibition and Induction of CYP3A4 on Cytotoxicity of DTN in PHH

**FIGURE 4:**
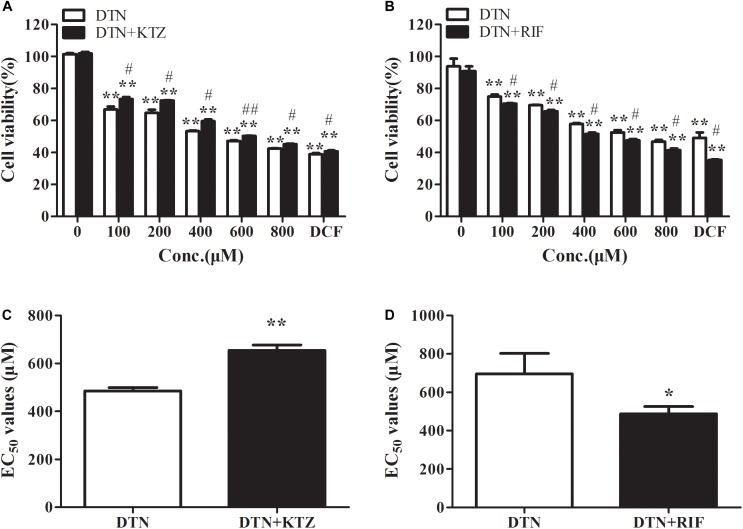
The effect of inhibition and induction of CYP3A4 on the cytotoxicity of DTN in PHH pretreated with KTZ **(A,C)** and RIF **(B,D)**, respectively. Data are represented as mean ± SD (*n* = 3). ^∗^*p* < 0.05, ^∗∗^*p* < 0.01 vs. control group; ^#^*p* < 0.05, ^##^*p* < 0.01 vs. DTN group.

As shown in **Figure 4A**, in the presence of KTZ, the cell viability of PHH was remarkably increased when treated with 1 mM DCF (positive control) for 24 h. Within the DTN concentration of 100–800 μM, the cytotoxicity was exacerbated and appeared to be dose-dependent. When treated with DTN+KTZ (10 μM), the cell viabilities at all tested concentrations showed significant decreases compared to the solvent group. The cytotoxicity of DTN (100–800 μM) co-treated with KTZ was statistically significant decreased by 2.63–8.65% in comparison with the DTN sole group. Moreover, the EC_50_ value was significantly increased in the KTZ pretreated group (**Figure 4C**).

The cell viability of PHH pre-incubated with RIF was significantly decreased when treated with 1 mM DCF (**Figure 4B**). Cytotoxicity of both DTN and DTN+RIF groups were significantly decreased compared with solvent control. At 100–800 μM of DTN, co-treatment with RIF resulted in a statistically significant decrease in cell viability (by 3.91–6.23%) and EC_50_ value compared to the DTN group (**Figure 4D**). In brief, when the activity of CYP3A4 was inhibited by KTZ *in vitro*, the cytotoxicity of DTN was alleviated, and once induced by RIF, the cytotoxicity was exacerbated.

### The Effect of Over-Expression of CYP3A4 on the Cytotoxicity of DTN in HepG2 and L02 Cells

**FIGURE 5:**
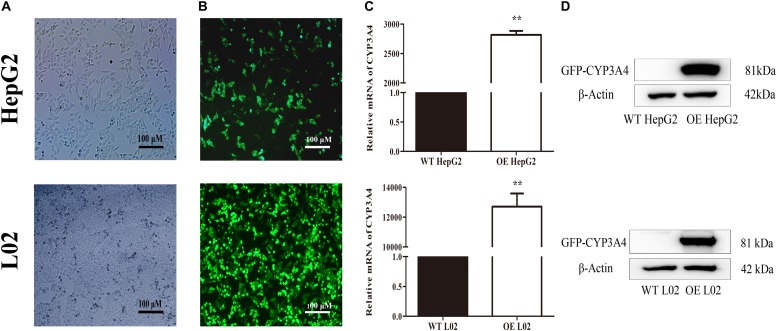
Characterization of wild type (WT) and stable CYP3A4 over-expressed (OE) HepG2 and L02 cells through **(A)** monitoring under white light, **(B)** monitoring under fluorescent light, **(C)** PCR and **(D)** Western blot. β-actin served as loading control (Original magnification 100×; the scale bar is 100 μm). ^∗∗^*p* < 0.01 vs. WT cells.

**FIGURE 6:**
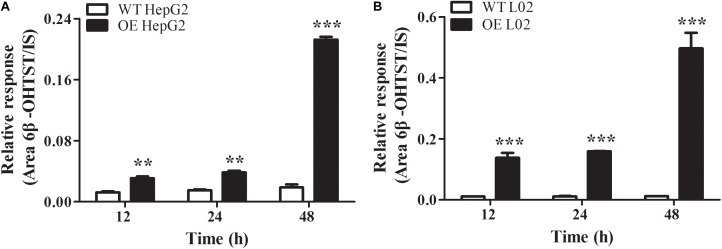
Oxidation of testosterone to 6β-hydroxyl-testosterone in WT and CYP3A4 OE HepG2 cells **(A)** and L02 cells **(B)**. Values are represented as mean ± SD (*n* = 3). ^∗^*p* < 0.05, ^∗∗^*p* < 0.01, ^∗∗∗^*p* < 0.001 vs. WT groups.

The stable CYP3A4 OE HepG2 and L02 cells were successfully built and verified. Both the OE cells showed green fluorescence under a fluorescence microscope (**Figures 5A,B**). The relative mRNA expression level of CYP3A4 in OE HepG2 and L02 cells was 2,700 and 13, 000 times higher than corresponding WT cells, respectively (**Figure 5C**). Both OE cells showed a GFP-CYP3A4 protein band simultaneously (**Figure 5D**). Furthermore, the enzyme activity of CYP3A4 was characterized through detecting the amount of 6β-hydroxyl testosterone in the respective cells incubating with testosterone (the classic substrate of CYP3A4) (**Figure 6**), which demonstrated that stable CYP3A4 OE cells were successfully constructed. The cytotoxicity comparison of DTN between CYP3A4 OE HepG2, L02 cells and the corresponding WT cells was performed. In addition, inhibition effect of KTZ on the OE cells was also explored. According to the cell viability and time curves, incubation for 48 and 24 h in OE HepG2 and L02 cells was, respectively, selected (**Supplementary Figure S1**).

**FIGURE 7:**
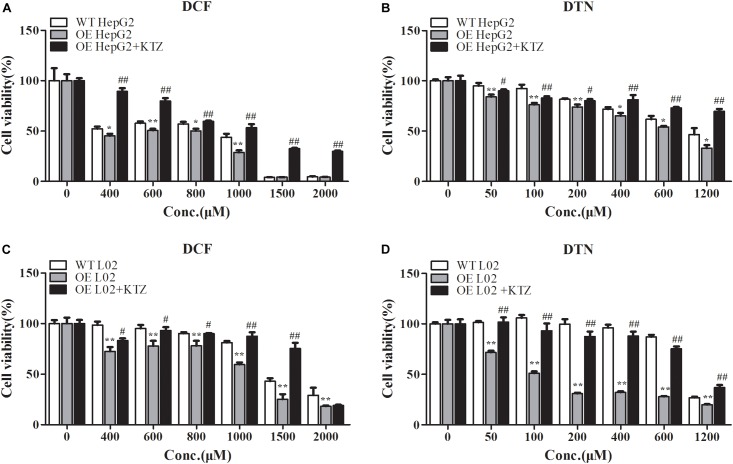
The effect of over-expression and inhibition of CYP3A4 on the cytotoxicity of DCF and DTN in HepG2 and L02 cells. **(A,B)** Over-expression and inhibition of CYP3A4 on the cytotoxicity of DCF and DTN in WT and OE HepG2 cells. **(C,D)** Over-expression and inhibition of CYP3A4 on the cytotoxicity of DCF and DTN in WT and OE L02 cells. Data are represented as mean ± SD (*n* = 3). ^∗^*p* < 0.05, ^∗∗^*p* < 0.01 vs. WT groups; ^#^*p* < 0.05, ^##^*p* < 0.01 OE+KTZ groups vs. OE groups.

Compared with WT cells, the cell viabilities in CYP3A4 OE HepG2 and L02 cells were greatly decreased when treated with DCF (400–2000 μM), and the decreased cell viability in both OE cells could be reversed by KTZ, which demonstrated that the model was suitable to validate the impact of CYP3A4 on the cytotoxicity (**Figures 7A,C**). When treated with DTN, the cell viability in OE HepG2 cells was significantly lower compared with WT HepG2 cells with the decrease ranging from 6.7 to 13.7% (**Figure 7B**). Furthermore, the cytotoxicity in OE HepG2 cells pretreated with KTZ was significantly decreased with the increase of cell viability from 5.9 to 36.8% compared with the OE cells. Meanwhile, the MTT assay showed that the cell viability in OE L02 cells was indeed decreased compared with WT cells after 24 h of DTN exposure, with decrease of cell viabilities ranging from 7.1 to 68.8% (**Figure 7D**). Similarly, co-incubation with KTZ could mitigate the cytotoxicity in OE L02 cells caused by CYP3A4 over-expression with cell viability increasing from 17.1 to 56.5%. Therefore, DTN elicited significantly higher cytotoxic effects in the CYP3A4 over-expressed HepG2 and L02 cells, and inhibition of CYP3A4 alleviated the cytotoxicity, which further revealed that CYP3A4 modulated DTN-induced toxicity.

### The Effect of Inhibition and Induction of CYP3A4 on Mouse Liver/Body Weight Ratio, Serum Parameters and Liver Histopathology

**FIGURE 8:**
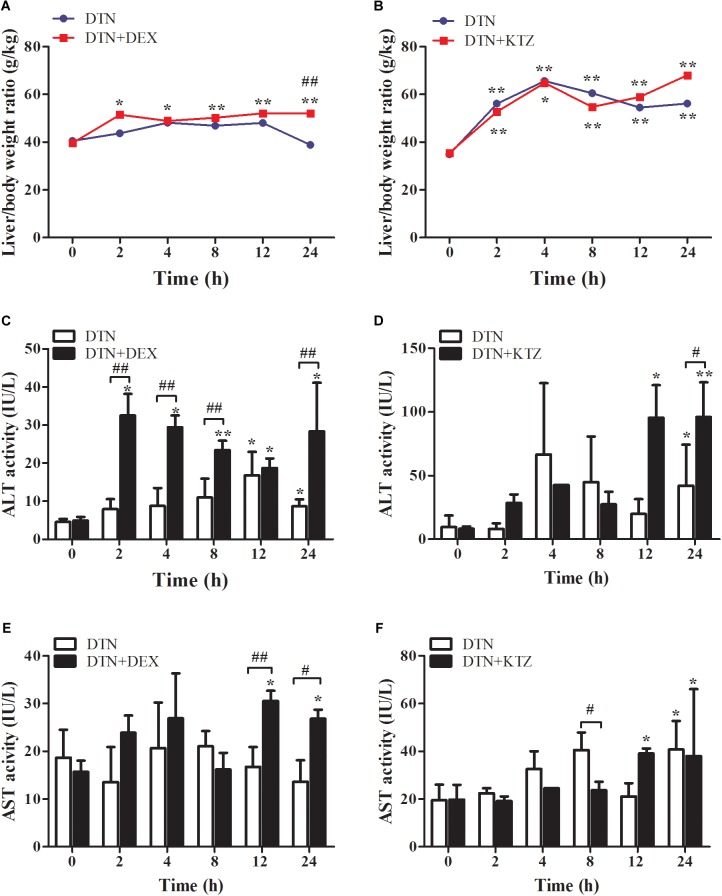
The effect of induction and inhibition of CYP3A4 on DTN-induced liver injury in mice. **(A,C,E)** Liver/body weight ratio, ALT and AST activities after exposure to DTN (a single dose of 640 mg/kg) in mice pretreated with DEX. **(B,D,F)** Liver/body weight ratio, ALT and AST activities after exposure to DTN (320 mg/kg for 4 days and 640 mg/kg on the 5th day) in mice pretreated with KTZ. Values are represented as mean ± SD (*n* = 4). ^∗^*p* < 0.05, ^∗∗^*p* < 0.01 vs. control group; ^#^*p* < 0.05, ^##^*p* < 0.01 vs. DTN group.

It is well known that metabolic enzymes vary among species. CYP3A11, the isoenzyme of human CYP3A4 in mouse, which occupies the amount of 20% of CYPs, is mainly involved in the metabolism of endogenous and exogenous compounds (Chen et al., 2018). The effect of DEX or KTZ on mice CYP3A11 was verified by Q-PCR and Western blot analysis. As shown in **Supplementary Figure S2** that the relative mRNA and protein expression level of CYP3A11 in DEX group was significantly increased (about 39%), and vice versa in KTZ group with about 20% decrease in CYP3A11 expression. Meanwhile, DTN treatment had no effect on the CYP3A11 expression. The results showed that the induction or inhibition models were successfully constructed. As shown in **Figure 8A**, oral administration of 640 mg/kg DTN with pretreatment of DEX significantly enlarged the liver/body weight ratio compared with the vehicle (at all time-points) and DTN sole group (at 24 h) In addition, In addition, ALT activity was significantly higher at 12 and 24 h in DTN group. And ALT activity in the DTN+DEX group was the greatest at 2 h, followed by a slight decrease from 4 to 12 h, and then was significantly raised at 24 h compared with DTN sole group (**Figure 8C**). Meanwhile, the AST activity was higher at 12 and 24 h with significance in DTN+DEX group (**Figure 8E**). Nevertheless, pretreatment with KTZ did not significantly decrease the liver/body weight ratio and ALT activity (**Figures 8B,D**), except that AST level was significantly decreased after 8 h exposure in DTN+KTZ group compared with DTN group (**Figure 8F**).

**FIGURE 9:**
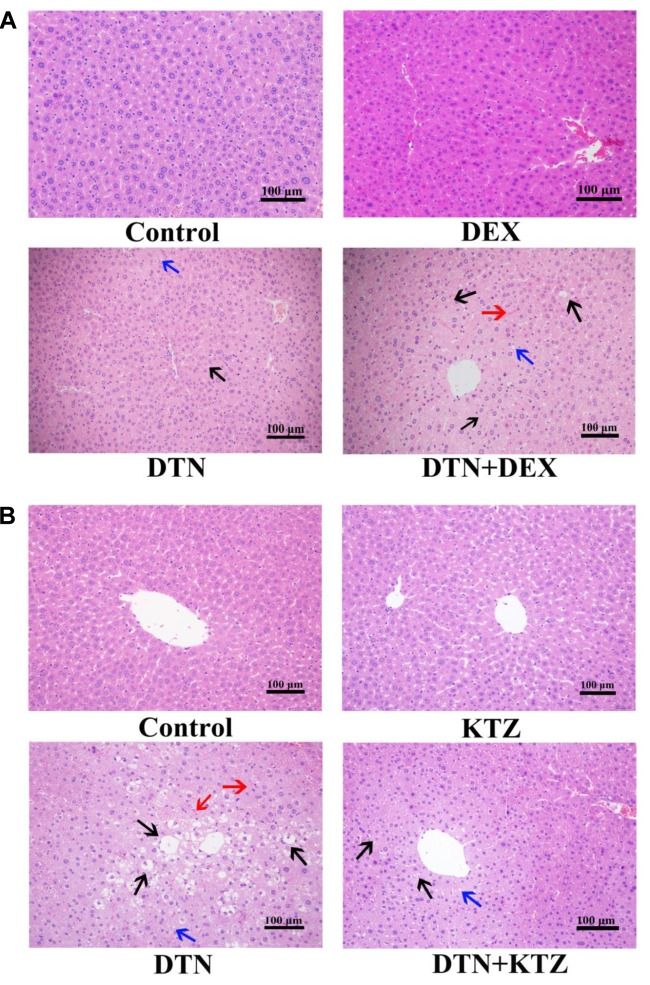
Histopathological evaluation (H&E staining) of liver tissues obtained from mice. Typical images were chosen from each experimental group. **(A)** Livers from mice exposed to DTN (a single dose of 640 mg/kg) in combination with DEX (70 mg/kg) and vehicle. **(B)** Livers from mice exposed to DTN (320 mg/kg for 4 days and 640 mg/kg on the 5th day) in combination with KTZ (75 mg/kg, 1.5 h before the last administration) and vehicle. (Original magnification 100×; the scale bar is 100 μm).

To supplement serum parameters, histopathological examination was performed for the DTN treatment group as well as vehicle controls. In group A, H&E evaluations suggested that administration of 640 mg/kg DTN caused slight hepatocyte degeneration whereas the livers of control mice appeared normal (**Figure 9A**). Mice pretreated with DEX, presented more serious liver injuries with medium hepatocyte degeneration (mainly edema and fatty degeneration) and several even showed slight congestion and inflammatory cell infiltration (labeled with arrows), compared with the mice treated with DEX alone (**Figure 9A**). In group B, pretreatment with KTZ before exposure to DTN mitigated liver injury with less degenerated hepatocytes and congestion cells, compared with the mice exposure to vehicle (**Figure 9B**). The detailed scores on the liver histopathology in groups A and B were listed in **Supplementary Tables S5**, **S6**. The histopathological results in combination with biochemical evaluations indicated that KTZ inhibition alleviated injury in mice, whereas, DEX induction aggravated the damage.

### Pharmacokinetic of DTN

**FIGURE 10:**
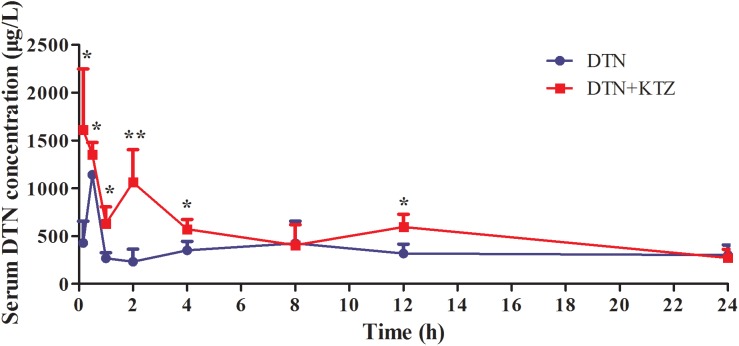
Time-course of measured DTN serum concentrations in mice following oral administration of DTN (320 mg/kg for 4 days and 640 mg/kg on the 5th day) with or without KTZ (75 mg/kg) pretreatment before the last administration. Data are represented as mean ± SD (*n* = 4). ^∗^*p* < 0.05, ^∗∗^*p* < 0.01 vs. DTN group.

Liquid chromatography-tandem mass spectrometry method was developed and fully validated, which met the requirement of Food and Drug Administration Guidance for Industry: Bioanalytical Method Validation for the determination of DTN in mice biological matrices (**Supplementary Figure S3** and **Supplementary Tables S2**–**S4**). The method was applied to investigate the pharmacokinetic parameters of DTN in mice, including *C*_max_, *T*_max_, AUC, *t*_1/2_, and V*z*/F and CL*z*/*F* with and without KTZ pretreatment, and the parameters were listed in **Table 2**. Significantly increased AUC (1.54-fold) and higher *C*_max_ (1.58-fold) were observed in mice pretreated with KTZ (**Figure 10**).

**Table 2:** Summary of DTN pharmacokinetic parameters obtained in mice with and without pretreatment of KTZ after oral administration of DTN (320 mg/kg for 4 days and 640 mg/kg on the 5th day, *n* = 4).

Parameter	DTN alone	DTN + KTZ
*C*_max_ (μg/L)	1162.11 ± 1003.18	1841.40 ± 462.34*
*T*_max_ (h)	0.88 ± 0.75	0.70 ± 0.88
AUC_0-*t*_ (μg/L^∗^h)	7028.68 ± 1992.49	10801.73 ± 3347.78*
AUC_0-∞_ (μg/L^∗^h)	14973.15 ± 7375.77	17557.64 ± 5722.71*
*t*_1/2_ (h)	19.67 ± 11.63	18.53 ± 10.14
CL*z*/F (L/h/kg)	0.03 ± 0.02	0.02 ± 0.01
V*z*/F (L/kg)	0.60 ± 0.15	0.53 ± 0.39


**^∗^Significantly different from mice treated with DTN alone (p < 0.05)*.*

## Discussion

The distribution of furan-containing natural products was extensive in CHMs. Despite of the beneficial effects some furan-containing natural products brought about, cases of hepatotoxicity related to those compounds are daily on the increase and should not be neglected. Actually, the hepatotoxicity of furan-containing compounds, such as teucrin A (Druckova et al., 2007), 4-ipomeanol (Bear et al., 2006), aflatoxin B1 (Larsson and Tjallve, 1992) and toosendanin (Yu et al., 2014), had been attributed to the metabolic activation of furan ring. A previous study found that CYP3A4 was the metabolic enzyme mediating bioactivation of the furan ring in toosendanin and yielded a *cis*-butene-1,4-dial intermediate (Yu et al., 2014). Considering the molecular structure of DTN possessing a furan ring and was primarily metabolic activated by CYP3A4 (Feng et al., 2016), the present study attempted to define the role of CYP3A4 in DTN-induced hepatotoxicity.

*In vitro* cytotoxicity of DTN was evaluated in PHH, HepG2, L02, and NIH3T3 cells. PHH remain differentiated, simultaneously sustain the major drug-metabolizing enzyme activities for a relatively long period of time in culture and afford the most similar excised environment to *in vivo* environment (LeCluyse, 2001). Simultaneously, the HepG2 and L02 cell lines have been considered a valuable model and are used for risk assessment of toxicants because they retain several liver functions (Rudzok et al., 2010), despite the lower activities of certain drug-metabolizing enzymes in comparison with PHH, such as CYP3A4 and CYP2C9 (Westerink and Schoonen, 2007). However, NIH3T3 cells, derived from mouse embryonic fibroblasts, are lack of CYPs expression (Xiao et al., 2014; Jiang J.Z. et al., 2017). In PHH, the EC_50_ value of DTN was 0.31 mM, which was 1.8 and 2.5 times smaller than that determined in L02 and HepG2 cells, respectively. In addition, DTN was almost non-toxic in NIH3T3 cells with free CYP3A4 expression presented as the irregular cytotoxicity ranging from 74.4 to 94.7% at different concentrations. Moreover, the cell viability was slightly decreased until up to 72 h incubation to evaluate the EC_50_ values. Toxicities expressed with EC_50_ values in PHH, L02, HepG2, and NIH3T3 cells were consistent with CYP3A4 expression levels in those cells, indicating that cytotoxicity required the participation of CYP3A4.

Furthermore, PHH were co-treated with the common *in vitro* specific inhibitor (KTZ) (Zahno et al., 2010) and inducer (RIF) of CYP3A4 (Zahno et al., 2011) to investigate the effect of CYP3A4 on DTN-induced cytotoxicity. It has been reported that a known hepatotoxic drug, DCF, caused hepatotoxicity through metabolic activation by CYP3A4. Therefore, DCF was chosen to serve as the positive control in this experiment. Theoretically, inhibition of CYP3A4 could mitigate cytotoxicity and induction aggravated cytotoxicity. The cell viability in the DCF+KTZ group was increased compared to the DTN group, presented as the increased EC_50_ value; while opposite results were observed in the RIF group, which was consistent with our speculation. With regard to DTN, all tested concentrations (100–800 μM) significantly increased cell viability after pretreatment with KTZ, and induction with DEX notably decreased the cell viability. It could be assumed that CYP3A4 plays a important role in the toxicity of DTN.

To verify the relationship between CYP3A4 and toxicity in depth, CYP3A4 OE HepG2 and L02 cells were established and certificated by measuring the expression of CYP3A4 on the mRNA and protein levels, as well as determination of the CYP3A4 activity. Results showed that the mRNA level was significantly higher in both the OE cells, and the metabolic activity in OE HepG2 and L02 cells was 3–11 times and 13–41 times significantly higher than in the respective WT cells (**Figure 6**). Comparison of cytotoxicity in OE and WT cells was performed based on the successfully established cells. The cytotoxicity in OE HepG2 and L02 cells was concentration-dependent, and the cell viabilities in OE cells treated with DCF and DTN both showed a significant decrease at tested concentrations compared with the corresponding WT cells. Additionally, the decrease in both the OE cells could be reversed by inhibition of CYP3A4 with KTZ. In combination with the results in PHH, it is evident that the up- and down-regulation of CYP3A4 were involved in the development of toxicity induced by DTN metabolic activation, namely high expression of CYP3A4 aggravated the cytotoxicity, and vice versa. To address the role of enzyme in detail, *in vivo* experiments in mice were further performed.

The recommended dosage of DC is 83–167 mg/kg/day for a 60 kg adult according to the Chinese Pharmacopoeia (Chinese Pharmacopoeia Commission, 2015). However, the clinical application of DC in prescription has been recently questioned, and it has been suggested that the therapeutic dose should be 250–833 mg/kg/day (Guo and Zhang, 2005; Wang et al., 2014). Accordingly, the equivalent dose for DTN in mice is approximately 0.9–3.0 mg/kg daily based on the content of DTN (0.03%) in DC (Yang et al., 2010). On account of the fact that DC related liver injury was a result of a long term and over-dose use, herein, in order to make acute toxicity models for single dose in this study, DTN was orally administrated at doses of approximately 80 times (320 mg/kg) and 160 times (640 mg/kg) higher than the highest dose used in clinic. Because CYP3As in the gut wall also have the potential to metabolize DTN, which may limit the understanding of the important role of hepatic CYP3As in the metabolism of DTN, it is justifiable to make the bioavailability and distribution of DTN after oral administration clear. As demonstrated (Wang et al., 2013), the absolute bioavailability of DTN from oral administration was determined as 47.9%, suggesting a very high proportion of DTN was absorbed into body’s circulation. Furthermore, the results on distribution showed that DTN was mostly distributed in the liver (twofold higher than in small intestine) after a rapid absorption. Although it is possible that the gut microbiota and intestinal P450 enzymes partially contribute to the metabolism of DTN, the above evidence suggested that DTN was mainly metabolized in liver. It is well known that ALT and AST activity are reliable and sensitive indicators of hepatocellular type of liver injury, as well as alkaline phosphatase (ALP) activity is a biomarker for cholestasis and bile duct injury (Ramaiah, 2007). Administration of DTN until at a dose of 640 mg/kg elevated ALT and AST levels in mice, but did not change ALP levels (**Supplementary Figures S4**, **S5**). The alterations in biochemical parameters were in accordance with symptoms in cases of DC-induced liver injury, which were clinically diagnosed as hepatocellular type instead of cholestatic type of HILI (Lee et al., 2015).

Moreover, we further established induction model or a repeated administration model for inhibition experiments in mice. The relative mRNA expression and hepatic CYP3A11 expression were up-regulated by potent inducer DEX (**Supplementary Figure S2B**). Mice pretreatment with DEX for 3 consecutive days as induction model were established. Changes in liver/body weight ratio were used as a direct indicator of liver injury, and pretreatment with DEX significantly increased liver/body weight ratio at 24 h. Furthermore, ALT level was significantly elevated within 24 h, except a slight decrease at 8 h exposure of DTN. Meanwhile, AST level was significantly elevated up to 24 h in DEX group. In addition, more severe histopathological injury with medium hepatocyte degeneration, slight congestion and inflammatory cell infiltration was observed in the DEX group, which indicated that induction of CYP3A4 aggravated liver injury.

On the contrary in the inhibition experiment, mice orally administrated with DTN at a dose of 320 mg/kg for 4 days and 640 mg/kg on the 5th day resulted in hepatocellular degeneration and congestion. However, no change of CYP3A11 expression was observed in mice treated with DTN (**Supplementary Figure S2C**), which might be due to the short time of administration. Moreover, a down-regulation of CYP3A11 expression (**Supplementary Figure S2C**) and mild toxic symptoms were observed in mice pretreated with KTZ on the 5th day, including a decrease in serum AST level and alleviation of hepatic degeneration compared with other regimen. Interestingly, the inconspicuous decrease of ALT and AST levels in KTZ group may be contributed to the fact that the biochemical factors are more sensitive, inhibition by KTZ presented a decrease of ALT and AST after 4 h exposure to DTN, and it lasted to 8 h. Afterwards, not only the debilitating down-regulating effect of KTZ but also the self-regulation of body brought about the dynamic recovery of ALT and AST level. Nevertheless, the liver histopathological response was slower and more reflective of the injury that inhibition by KTZ could mitigate hepatocellular degeneration and congestion to some extent. Therefore, the *in vivo* results in depth illustrated that CYP3A4 participated in and modulated DTN-induced toxicity.

Due to the fact that DTN could be metabolized via CYP3A4 (phase I metabolism), the change in CYP3A4 expression might not only influence the phase I metabolism of DTN, but also alter serum concentration of DTN as well. Therefore, the serum concentration of DTN was measured by LC-MS/MS. Pharmacokinetic data showed larger AUCs and higher *C*_max_ of DTN in serum of DTN+KTZ group compared with DTN group alone, which suggested that the inhibition of CYP3A4 led to the enhancement of absorption of DTN *in vivo*. However, higher *C*_max_ and larger AUCs of DTN were not accompanied by more potent hepatotoxicity (**Figures 8F**, **9B**), that is to say mice pretreated with KTZ were less susceptible to DTN-induced hepatotoxicity, which further implicated that CYP3A4 participated in the process of DTN-induced hepatotoxicity and metabolic intermediate rather than the parent drug was likely to exert the toxicity. There is a serious concern that multiple clinically relevant drug interactions with CHMs can occur in HILI cases (Teschke et al., 2012, 2013). Herb–drug interactions may change the available affinity of specific receptor or systemic pharmacokinetic actions of drug, including absorption, distribution, metabolism and excretion. Therefore, the present study offers valuable reference when DC is clinically prescribed for combination therapy.

## Conclusion

In summary, exposure to DTN exhibited liver injury in both cells and mice. Induction of CYP3A4 aggravated toxic effects *in vitro* and *in vivo*, whereas inhibition of CYP3A4 attenuated the hepatotoxicity. This work provides insights into the role of CYP3A4 in the DTN-induced liver injury, which would be helpful to comprehensively illustrate the relationship between the toxic actions of DTN and metabolic activation. However, the relative weak, albeit statistically significant effects of induction/inhibition and over-expression of CYP3A4 on DTN cytotoxicity suggest that non-CYP3A4 drug metabolizing enzyme may also play a role in DTN-induced toxicity. Further study on detecting the metabolic activated intermediates, thoroughly characterizing the DTN metabolic activated adducts *in vivo* and *in vitro* and ascertain the relationship between adduct and hepatotoxicity should be performed.

## Author Contributions

H-JL and PL conceived and designed the experiments. Z-QL, L-LJ, and D-SZ performed the animal experiments. Z-QL and JZ analyzed the data. L-LW, Z-TW, and XZ contributed reagents and materials analysis. H-JL and Z-QL wrote the manuscript. Z-QS revised the manuscript and contributed the pharmacokinetic study.

## Conflict of Interest Statement

The authors declare that the research was conducted in the absence of any commercial or financial relationships that could be construed as a potential conflict of interest.
