# Supplementary material for: The Modulatory Role of CYP3A4 in Dictamnine-Induced Hepatotoxicity
Source: Front Pharmacol. 2018 Sep 19;9:1033. doi: 10.3389/fphar.2018.01033 (PMC6156363; doi:10.3389/fphar.2018.01033)
Supplement: Supplementary file 1 [file Data_Sheet_1.docx]

**Supplementary material**

**The crucial modulatory role of CYP3A4 in dictamnine-induced hepatotoxicity**

Zhuo-Qing Li^a^, Li-Long Jiang^a^, Dong-Sheng Zhao^a^, Jing Zhou^a^, Ling-Li Wang^a^, Zi-TianWu^a^, Xian Zheng^a^, Zi-Qi Shi ^b,c^*, Ping Li^a^, Hui-Jun Li^a,^*

*^a^* State Key Laboratory of Natural Medicines, China Pharmaceutical University, No. 24 Tongjia Lane, Nanjing, China

*^b^* Affiliated Hospital of Integrated Traditional Chinese and Western Medicine, Nanjing University of Chinese Medicine, Nanjing, China

*^c^* Jiangsu Province Academy of Traditional Chinese Medicine, Nanjing, China

**Correspondence:**

Hui-Jun Li, PhD

State Key Laboratory of Natural Medicines, China Pharmaceutical University, No. 24 Tongjia Lane, Nanjing 210009, China.

E-mail: cpuli@163.com.

Tel.: +86 25 83271382; Fax: +86 25 83271379.

Zi-Qi Shi, PhD,

Key Laboratory of New Drug Delivery Systems of Chinese Materia Medica, Jiangsu Provincial Academy of Chinese Medicine, Nanjing 210028, China

E-mail: shiziqi47@126.com.

Tel.: +86 25 85608672, fax: +86 25 85608672.

**METHODS**

**Effect of Incubation Time on Cytotoxicity of DTN in HepG2 and L02 Cells**

HepG2 and L02 cells were seeded into a 96-well plate at a density of 1 × 10^4^ cells per well, incubated for 24 h, and then exposed to a blank control, DTN (100 – 1200 μM) and DMSO. After incubation for 6, 24 and 48 h, The MTT assay was employed and the optical density was measured at 490 nm with a microplate reader (BioTek Instruments, Winooski, VT, USA). As shown in Supplementary Figure 1, cytotoxicity in HepG2 cells was concentration- and time-dependent, while the time-dependent trend in L02 cells was not apparent. Therefore, incubation for 48 and 24 h was selected for both wild type and CYP3A4 over-expressed HepG2 and L02 cells, respectively.

**Verification of Expression of CYP3A11 Pretreated with DEX or KTZ in Mice**

20 mg of each mouse liver sample was weighed, and homogenized with 1 mL trizol in the condition of 60 Hz for 30 s in 4 °C. The supernatant was separated to extract the RNA. Other procedures were the same as above except for using the primes of mice. Human and mouse GAPDH were used as the corresponding control. The primers (Supplementary Table 1) were from Genscript Biotech Co., Ltd. (Nanjing, China). For the western bolt analysis, liver homogenates were prepared in RIPA (radio immunoprecipitation assay) lysis buffer. Protein concentrations were determined using BCA assay reagents according to the manufacturer’s instructions. Other procedures were the same as in the manuscript except for using CYP3A11 antibody (Millipore, Temecula, CA, USA; Lot No: 3027080). The gray values of the bands were analyzed by the alphaEaseFC software (Alpha Innotech, USA). The result was shown in Supplementary Figure 2A that pretreatment with DEX increased the mRNA expression of CYP3A11 and vice versa in KTZ group. Western blot analysis showed that hepatic CYP3A11 expression was up-regulated by about 39% in DEX group (Supplementary Figure 2B). However, no change of CYP3A11 expression was observed in mice treated with DTN and a down-regulation of CYP3A11 expression (about 20%) was observed in the KTZ pretreatment group (Supplementary Figure 2C).

**Establishment and Validation of LC-QqQ-MS Method**

**Selectivity**

The selectivity was investigated by analyzing blank mouse serum, blank mouse serum spiked with analyte and IS, and actual mouse serum sample after oral administration of DTN, respectively. Representative chromatograms were shown in Supplementary Figure 3, no endogenous interfering peaks were observed in the serum sample at the measured mass transitions and retention times of the analyte and IS for the highly selective MRM mode.

**Linearity and LLOQ**

The linearity was evaluated by plotting the peak area ratio of analyte to IS (y) against the corresponding concentration of analyte (x). Least square linear regression analysis with a weighting factor of 1/C^2^ was applied to determine the slope, intercept and correlation coefficient. The LLOQ was determined to be the lowest concentration on the calibration curve with an acceptable precision and accuracy within 20%. The calibration curve were obtained over the concentration range from 0.4 to 1000 ng/mL for DTN, and exhibited good linearity (*r*^2^ = 0.9996). The LLOQ for DTN was 0.4 ng/mL. The method was sensitivity and satisfactory for the pharmacokinetics study of DTN.

**Precision and accuracy**

Intra-day and inter-day precision and accuracy were also assessed by analyzing five replicates of QC samples (low, medium, and high concentration levels) for DTN on the same day and on three consecutive days. The precision and accuracy were expressed as relative standard deviation (RSD, %) and relative error (RE, %), respectively. The results of intra- and inter-day precision and accuracy were summarized in Supplementary Table 2. Intra-day and inter-day precisions of DTN ranged from 0.01% to 2.64%, and from 0.36% to 4.53%, while the accuracies of DTN ranged from −6.26% to 13.76%, and from −5.89% to −3.39%, respectively. The results indicated that the precision and accuracy of this method were acceptable.

**Extraction Recovery and Matrix Effect**

The extraction recoveries of DTN at three QC levels were determined by comparing mean peak areas obtained from samples that the DTN were added prior-extraction, with those spiked in post-extraction at the same concentration. The matrix effect was determined by comparing the relative peak areas obtained from blank mice serum extract spiked with analyte with those of standard solutions at the same concentration.

Results are presented in Supplementary Table 3. The recoveries of DTN ranged from 87.96% to 91.75% at three concentration levels. This demonstrated that the [sample preparation](https://www.sciencedirect.com/topics/biochemistry-genetics-and-molecular-biology/sample-preparation) method could ensure the acquisition of accurate and consistent data. The matrix effect was between 87.45% and 109.87%, suggesting that there was no significant ion suppression in this method.

**Stability**

The stabilities of analytes in spiked mice serum were evaluated for five replicates of QC samples under different conditions. Short-term and long-term stability were assessed by placing QC samples at room temperature for 6 h and at −20 °C for 14 days. The post-preparation stability was tested by keeping QC samples in the auto-sampler at 4 °C for 24 h. The freeze-thaw cycle stability was determined after three freeze-thaw cycles (from −80 °C to 25 °C) on three consecutive days. Results are listed in Supplementary Table 4. In general, the analytes remained stable under different storage conditions with RE ranged from −14.83% to −4.40% for 14 days at −20 °C, from −6.13% to 0.35% for 6 h at room temperature, and from −4.70% to 4.27% for 24 h at 4 °C in auto-sampler, respectively. The accuracy of freeze-thaw cycle stability was between −14.03% and −2.51%. The results showed that the established method was suitable for large scale sample analysis.

**The Dose and Time Effects on Mouse Serum Parameters after Oral Administration of DTN**

To assess DTN toxicity, mice were treated with a single oral dose of DTN (4, 40, 80, 160, 320 and 640 mg/kg), thereby serum parameters of ALT, AST, ALP, TBA, TBIL, and BUN were examined. ALT and AST levels were elevated at 24 h after exposure, and serum parameters were significantly increased until 640 mg/kg. Additionally, ALP activity was increased at 320 mg/kg. The levels of TBA, TBIL and BUN in serum did not remarkably change after treatment with the various dosages of DTN except for a slight elevation in TBA that was observed in the 40 mg/kg group (Supplementary Figure 4). The drug disposition was dynamic, which manifested as varied biochemical parameters. As shown in Supplementary Figure 5, the ALT activity was significantly increased after 12 h of 640 mg/kg DTN exposure. Additionally, AST activity was twice that of the control in at 4 h, followed by a slight decrease at 12 and 24 h after administration of DTN. In addition, the ALP level was higher at 24 h. The results indicated that liver injury induced by DTN was time-independent.

**SUPPLEMENTARY FIGURES LEGENDS**

**Supplementary FIGURE 1. Cytotoxicity of DTN in HepG2 (A) and L02 cells (B) for different incubation time**

**Supplementary FIGURE 2. Effect of DEX and KTZ on the mRNA expression (A) and protein expression (B, C) of CYP3A11. ^*^*p* < 0.05 vs. control group.**

**Supplementary FIGURE 3. Representative chromatograms of DTN (1) and γ-fagarine (2, IS) in serum samples. (a) blank serum sample. (b) blank serum sample spiked with DTN and IS. (c) serum sample at 1 h after treated with 640mg/kg of DTN.**

**Supplementary FIGURE 4. Dose effect on serum ALT (A), AST (B), ALP (C), TBA (D), TBIL (E), and BUN (F) activities in mice exposed to a single dose of DTN and vehicle. Values are represented as mean ± SD (*n* = 6). ^*^*p* < 0.05, ^**^*p* < 0.01 vs. control group.**

**Supplementary FIGURE5. Time effect on serum ALT (A), AST (B), and ALP (C) activities in mice after exposed to a single dose of DTN (640 mg/kg) and vehicle. Values are represented as mean ± SD (*n* = 6). ^*^*p* < 0.05, ^**^*p* < 0.01 vs. control group.**

**Supplementary FIGURE 1. Cytotoxicity of DTN in HepG2 (A) and L02 cells (B) for different incubation time**


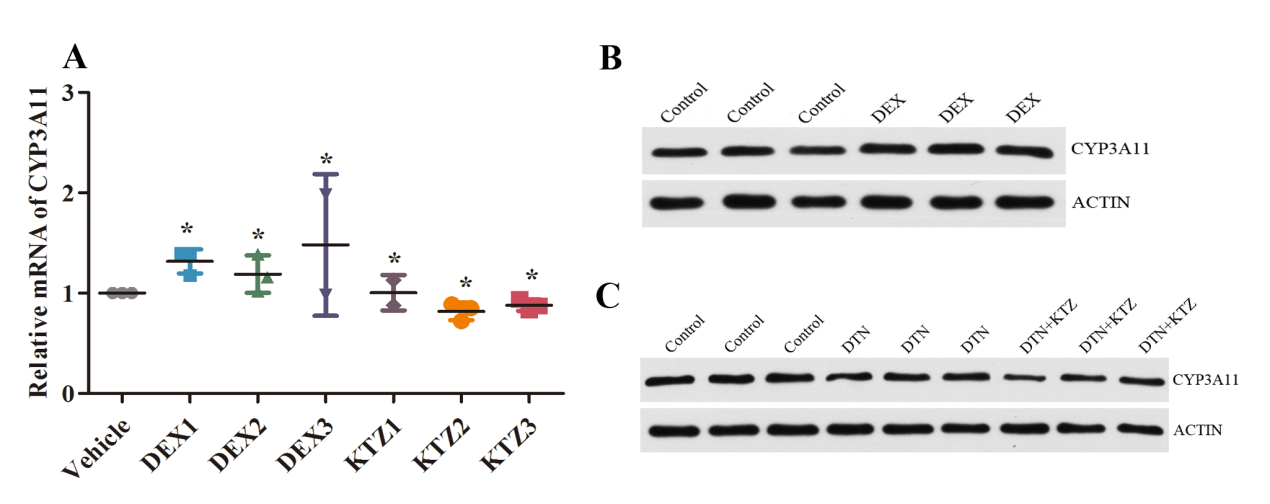


**Supplementary FIGURE 2.** **Effect of DEX and KTZ on the mRNA expression (A) and protein expression (B, C) of CYP3A11. ^*^*p* < 0.05 vs. control group.**


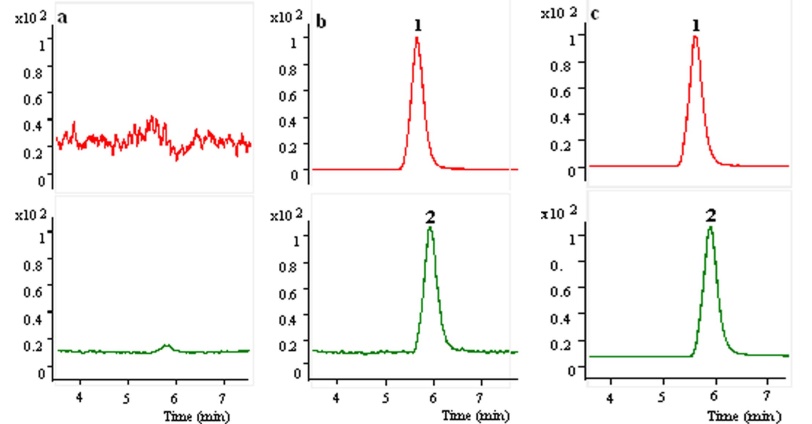


**Supplementary FIGURE 3. Representative chromatograms of DTN (1) and γ-fagarine (2, IS) in serum samples. (a) blank serum sample. (b) blank serum sample spiked with DTN and IS. (c) serum sample at 1 h after treated with 640mg/kg of DTN.**

**Supplementary FIGURE 4. Dose effect on serum ALT (A), AST (B), ALP (C), TBA (D), TBIL (E), and BUN (F) activities in mice exposed to a single dose of DTN and vehicle. Values are represented as mean ± SD (*n* = 6). ^*^*p* < 0.05, ^**^*p* < 0.01 vs. control group.**

**Supplementary FIGURE 5. Time effect on serum ALT, AST, and ALP activities in mice after exposed to a single dose of DTN (640 mg/kg) and vehicle. Values are represented as mean ± SD (*n* = 6). ^*^*p* < 0.05, ^**^*p* < 0.01 vs. control group.**

**Supplementary TABLE 1 Sequences of primer pair used for PCR amplification**

| Target genes | 5’→3’ primer sequences  (Forward) | 5’→3’ primer sequences  (Reverse) |
| --- | --- | --- |
| mGAPDH | CGTGCCGCCTGG  AGAAACCTG | AGAGTGGGAGTT  GCTGTTGAAGTCG |
| mCYP3A11 | ACAAACAAGCAG  GGATGGAC | GGTAGAGGAGCA  CCAAGCTG |

**Supplementary TABLE 2** **Intra- and inter-day precision and accuracy for the determination of DTN in mice serum (*n* = 5).**

| Analyte | Norminal con. (ng/mL) | Inter-day | | Intra-day | |
| --- | --- | --- | --- | --- | --- |
|  |  | Precision  (RSD, %) | Accuracy  (RE, %) | Precision  (RSD, %) | Accuracy  (RE, %) |
| DTN | 1 | 4.53 | −4.94 | 2.64 | 13.76 |
|  | 20 | 1.78 | −3.39 | 0.62 | −8.73 |
|  | 400 | 0.36 | −5.89 | 0.01 | −6.26 |

**Supplementary TABLE 3** **Matrix effect and extraction recovery for the determination of DTN in mice serum (*n* = 5).**

| Analyte | Spiked con.  (ng/mL) | Matrix effect  (%,mean±SD) | Extraction efficiency  (%,mean±SD) |
| --- | --- | --- | --- |
| DTN | 1 | 109.87±4.98 | 91.75±8.51 |
|  | 20 | 87.45±3.54 | 87.96±5.63 |
|  | 400 | 89.86±8.58 | 88.24±1.90 |

**Supplementary TABLE 4** **Stability of DTN in mice serum (*n* = 5)**

| Analyte | Norminal con.(ng/mL) | Ambient temperature  for 6h | | 4 °C for 24 h | | Three-freeze-thaw cycles | | Frozen for 14 days | |
| --- | --- | --- | --- | --- | --- | --- | --- | --- | --- |
|  |  | Measured con.(ng/mL) | Accuracy  (RE, %) | Measured con.(ng/mL) | Accuracy  (RE, %) | Measured  con.(ng/mL) | Accuracy  (RE, %) | Measured  con.(ng/mL) | Accuracy  (RE, %) |
| DTN | 1 | 1.00±0.09 | 0.35 | 1.04±0.14 | 4.27 | 0.86±0.10 | −14.03 | 0.85±0.13 | −14.83 |
|  | 20 | 19.20±0.53 | −3.98 | 19.14±1.20 | −4.29 | 19.50±0.92 | −2.51 | 19.12±1.13 | −4.40 |
|  | 400 | 375.49±10.29 | −6.13 | 381.19±11.47 | −4.70 | 378.19±6.28 | −5.45 | 374.92±7.88 | −6.27 |

**Supplementary TABLE 5 Histopathologic microscopic examination of liver H & E sections in Group A**

**(Mice was exposed to a single dose of DTN in combination with DEX (70 mg/kg) and vehicle)**

**^*^*p* < 0.05 vs. control group, ^#^*p* < 0.05 DTN+DEX vs. DTN group.**

| **Groups** | **Numbers** | **Congestion** | **hepatocytes** | | | **Inflammatory cells** | **Scores** |
| --- | --- | --- | --- | --- | --- | --- | --- |
|  |  |  | **Degeneration** | **Necrosis** | **Glycogen deposition** |  |  |
| Control | 1 |  |  |  |  |  | 0 |
|  | 2 |  |  |  |  | 0.5 | 0.5 |
|  | 3 |  | 0.5 |  |  |  | 0.5 |
|  | 4 |  |  |  |  |  | 0 |
| **n = 4 mean ± SD = 0.25 ± 0.29** | | | | | | | |
| DTN | 1 |  | 1 |  |  |  | 1 |
|  | 2 |  | 0.5 |  |  |  | 0.5 |
|  | 3 |  | 1 |  |  |  | 1 |
|  | 4 |  | 1 |  |  |  | 1 |
| **n = 4 mean ± SD = 0.88 ± 0.25^*^** | | | | | | | |
| DEX | 1 |  |  |  |  |  | 0 |
|  | 2 | 1 |  |  |  |  | 1 |
|  | 3 |  |  |  |  |  | 0 |
|  | 4 |  |  |  |  |  | 0 |
| **n = 4 mean ± SD = 0.25 ± 0.50** | | | | | | | |
| DTN+DEX | 1 | 0.5 | 1 |  |  |  | 1.5 |
|  | 2 |  | 1.5 |  |  | 0.5 | 2 |
|  | 3 |  | 2 |  |  |  | 2 |
|  | 4 |  | 2 |  |  |  | 2 |
| **n = 4 mean ± SD = 1.88 ± 0.24^#^** | | | | | | | |

**Supplementary TABLE 6 Histopathologic microscopic examination of liver H & E sections in Group B**

**(Mice was exposed to DTN (320 mg/kg for 4 days and 640 mg/kg on the 5^th^ day) in combination with KTZ (75 mg/kg) and vehicle)**

**^*^*p* < 0.05 vs. control group, ^#^*p* < 0.05 DTN+KTZ vs. DTN group.**

| **Groups** | **Numbers** | **Congestion** | **hepatocytes** | | | **Inflammatory cells** | **Scores** |
| --- | --- | --- | --- | --- | --- | --- | --- |
|  |  |  | **Degeneration** | **Necrosis** | **Glycogen deposition** |  |  |
| Control | 1 |  |  |  |  |  | 0 |
|  | 2 |  | 0.5 |  |  |  | 0.5 |
|  | 3 |  |  |  |  | 0.5 | 0.5 |
|  | 4 |  |  |  |  |  | 0 |
| **n = 4 mean ± SD = 0.25 ± 0.29** | | | | | | | |
| DTN | 1 | 0.5 | 1 |  |  |  | 1.5 |
|  | 2 |  | 0.5 |  |  |  | 0.5 |
|  | 3 |  | 1 |  |  |  | 1 |
|  | 4 |  | 0.5 | 0.5 |  |  | 1 |
| **n = 4 mean ± SD = 1.00 ± 0.40^*^** | | | | | | | |
| KTZ | 1 |  |  |  |  |  | 0 |
|  | 2 |  |  |  |  |  | 0 |
|  | 3 |  |  |  |  | 0.5 | 0.5 |
|  | 4 |  | 0.5 |  |  |  | 0.5 |
| **n = 4 mean ± SD = 0.25 ± 0.29** | | | | | | | |
| DTN+KTZ | 1 |  | 0.5 |  |  |  | 0.5 |
|  | 2 |  | 0.5 |  |  | 0.5 | 1 |
|  | 3 | 0.5 |  |  |  |  | 0.5 |
|  | 4 |  | Lipid droplets caviation |  |  |  | 0 |
| **n = 4 mean ± SD = 0.50 ± 0.41^#^** | | | | | | | |
